# Metabolite identification in fecal microbiota transplantation mouse livers and combined proteomics with chronic unpredictive mild stress mouse livers

**DOI:** 10.1038/s41398-017-0078-2

**Published:** 2018-01-31

**Authors:** Bo Li, Kenan Guo, Li Zeng, Benhua Zeng, Ran Huo, Yuanyuan Luo, Haiyang Wang, Meixue Dong, Peng Zheng, Chanjuan Zhou, Jianjun Chen, Yiyun Liu, Zhao Liu, Liang Fang, Hong Wei, Peng Xie

**Affiliations:** 1 Institute of Neuroscience and the Collaborative Innovation Center for Brain Science, Chongqing Medical University, Chongqing, China; 2 Chongqing Key Laboratory of Neurobiology, Chongqing, China; 3 Key Laboratory of Clinical Laboratory Diagnostics (Ministry of Education), Chongqing, China; 4 Department of Laboratory Animal Science, College of Basic Medical Sciences, Third Military Medical University, Chongqing, China; 5 Department of Neurology, Yongchuan Hospital, Chongqing Medical University, Chongqing, China; 6 Department of Neurology, The First Affiliated Hospital of Chongqing Medical University, Chongqing, China; 7 South Australian Health and Medical Research Institute, Mind and Brain Theme, Flinders University, Adelaide, SA Australia

## Abstract

Major depressive disorder (MDD) is a common mood disorder. Gut microbiota may be involved in the pathogenesis of depression via the microbe–gut–brain axis. Liver is vulnerable to exposure of bacterial products translocated from the gut via the portal vein and may be involved in the axis. In this study, germ-free mice underwent fecal microbiota transplantation from MDD patients and healthy controls. Behavioral tests verified the depression model. Metabolomics using gas chromatography–mass spectrometry, nuclear magnetic resonance, and liquid chromatography–mass spectrometry determined the influence of microbes on liver metabolism. With multivariate statistical analysis, 191 metabolites were distinguishable in MDD mice from control (CON) mice. Compared with CON mice, MDD mice showed lower levels for 106 metabolites and higher levels for 85 metabolites. These metabolites are associated with lipid and energy metabolism and oxidative stress. Combined analyses of significantly changed proteins in livers from another depression model induced by chronic unpredictive mild stress returned a high score for the Lipid Metabolism, Free Radical Scavenging, and Molecule Transports network, and canonical pathways were involved in energy metabolism and tryptophan degradation. The two mouse models of depression suggest that changes in liver metabolism might be involved in the pathogenesis of MDD. Conjoint analyses of fecal, serum, liver, and hippocampal metabolites from fecal microbiota transplantation mice suggested that aminoacyl-tRNA biosynthesis significantly changed and fecal metabolites showed a close relationship with the liver. These findings may help determine the biological mechanisms of depression and provide evidence about “depression microbes” impacting on liver metabolism.

## Introduction

Major depressive disorder (MDD) is a debilitating mental disorder accounting for 12.3% of the global burden of disease and affects up to 15% of the general population^1^. Recent studies suggest that MDD is associated with neurotrophic alterations^2^, an imbalance in the hypothalamic–pituitary–adrenal axis^3^, and glutamine neurotransmitter system dysfunction^4^. Most research on depression focuses on changes to the brain, and few studies have examined the effects of liver metabolism on depression.

Accumulating evidence suggests that disruption of gastrointestinal microbes is associated with the development or exacerbation of mental disorders in humans, and the microbe–gut–brain axis may play a key role in maintaining brain health and stress responses^5–7^.

A mouse model of depression has been established using fecal microbiota transplantation (FMT), in which fecal matter from MDD patients is implanted into germ-free mice^8,9^. The germ-free mouse, which is free from bacterial contamination, has been widely used to investigate interactions between the microbiota and host^10^.

The liver is the main organ for substrate and energy metabolism and plays an important role in oxidative stress, glycogen storage, and the synthesis of secretory proteins. A previous study reported that liver diseases were associated with depression and suicide attempts^11^.

Recently, metabolomics has been used to define metabolic disorders and specific biomarkers of various disease states^12,13^. We have applied metabolomics to the serum^14^, urine^15^, and peripheral blood mononuclear cells^16^ of MDD patients, and the prefrontal cortex of a lipopolysaccharide (LPS)-induced mouse model of depression^17^. During these studies, we identified five metabolites in urine from MDD patients uniquely produced by bacteria in the intestinal tract that were significantly decreased relative to healthy controls (CONs). Of note, some clinical studies have reported that MDD induced a relatively high incidence of irritable bowel syndrome, a disease involving gut microbe disorders^15,18^. It is possible that MDD has an association with intestinal microbe dysbiosis. Further, we analyzed fecal, serum, and hippocampal samples from a depression microbe-induced mouse model, and found that the metabolites might be associated with carbohydrate, amino acid, and nucleotide metabolism^9^. Based on these findings, we considered “depression microbes” can affects metabolism in the gut tract and serum. Whether microbiota dysbiosis impacts on the liver metabolism profile and whether the liver plays an important role in the microbe–gut–brain axis are not clear.

With diverse methodologies, the combination of different metabolomics platforms can identify more synthesis metabolites than a single method^19–21^. In the current study, three metabolomics approaches using nuclear magnetic resonance (NMR), gas chromatography–mass spectrometry (GC–MS), and liquid chromatography–mass spectrometry (LC–MS) were combined to determine metabolomics profile alterations in the livers of a mouse model of MDD.

## Materials and methods

### Ethical considerations

Animal experiments were approved by the Third Military Medical University (Chongqing, China) and the Ethical Committee of Chongqing Medical University (Chongqing, China). Kunming (germ-free) mice were obtained from the Experimental Animal Research Center at the Third Military Medical University and were kept in flexible film gnotobiotic isolators until 6 weeks old and weighing 30–40 g. Mice were housed in standard autoclaved polypropylene cages with access to food and water ad libitum under a 12-h dark–light cycle (light from 08:00 AM until 08:00 PM), and at a constant temperature (23 ± 1 °C) and relative humidity (50 ± 5%). Mice acclimatized for 2 weeks to standard experimental conditions prior to the commencement of experiments.

### FMT and sample collection

MDD patients were diagnosed following a structured psychiatric interview using DSM-IV-TR criteria and 17 items from the Hamilton depression rating scale. The FMT was carried out via randomly selecting 0.5 g of feces from five MDD or healthy individuals under an oxygen-free environment, and all 0.5 g samples were mixed in 7.5 mL of 0.9% saline to obtain suspension. Then the microbiota was transplanted into germ-free mice in a flexible film gnotobiotic isolator. After 2 weeks, behavioral tests were performed. On completion of behavioral tests, the livers of mice were immediately collected and stored at −80 °C until metabolism analysis.

### Gas chromatography–mass spectrometry

Twenty-four mouse livers (12 MDD and 12 CON) were prepared for GC–MS metabolomics analysis. Briefly, a 100-mg liver sample was derivatized with pyridine hydrochloride solution and N,O-bis(trimethylsilyl)trifluoroacetamide (including 1% trimethylchlorosilane) before undergoing GC–MS analysis. Full details for derivatization and GC–MS conditions have been reported^19^. Analysis used the exported NetCDF file format and TagFinder. As an internal standard, L-2-chlorophenylalanine (0.03 mg/mL; methanol configuration) was used to normalize peak areas of extracted ions.

### Nuclear magnetic resonance

Liver tissue (~ 100 mg) was mixed with 800 µL methanol–water (4:1, v/v) before being homogenized, ultrasonic extraction for 5 min, stewing for 20 min at 4 °C, and centrifugation at 14,000 g for 10 min at 4 °C. Supernatant (200 µL) was transferred to a glass bottle for rapid centrifugal concentrator volatile drying before being dissolved in 500 µL heavy water in a NMR tube prior to detection.

Proton spectra were detected using a Varian 600 spectrometer at an operating power of 599.925 MHz in ^1^H. A Carr–Purcell–Meiboom–Gill (recycle delay−90°−(τ−180°−τ)n−acquisition) pulse sequencer with a relaxation delay of 2.5 s, a mixing time of 100 m/s, a spectral width of 10 kHz, and a data point of 16 K was used cumulatively 128 times. Discrimination between MDD and CON mice was visualized using partial least-squares discriminant analysis. Coefficient loading plots of the model were used to identify the spectral variables responsible for the sample differentiation on the score plot. A correlation coefficient of │r│ > 0.553 based on a *p*-level < 0.05 or variable importance in projection (VIP) value > 1.000 was used as the cutoff value for statistical significance.

### Liquid chromatography–mass spectrometry

LC–MS preparation was performed as previously described^9^. Briefly, 100 mg of liver sample was homogenized with 20 µL internal standard (l-2-chloro-l-phenylalanine, 0.03 mg/mL; methanol configuration) and 800 µL methanol–water solution (4/1, v/v) before ultrasonic extraction for 5 min, incubation for 20 min at 4 °C, and centrifugation for 10 min at 14,000 g at 4 °C. Supernatant (200 µL) was transferred into a glass bottle for LC–MS metabolomics analysis. Supernatant underwent ultra-performance liquid chromatography–tandem mass spectrometry (UPLC-Q-TOF/MS). Mass spectrometric data were collected using a Waters VION IMS Q-TOF mass spectrometer equipped with an electrospray ionization source operating in either positive or negative ion mode. Full details are provided in a previous study^9^. Orthogonal partial least-squares discriminant analysis (OPLS-DA) was used to identify differential metabolites in MDD mice compared with CON mice.

### Metabolomics function and pathway analyses

For significantly altered metabolites (*p* < 0.05 and VIP > 1.0), pathway analyses was performed using MetaboAnalyst 3.0 (http://www.metaboanalyst.ca/) and Ingenuity pathway analysis (IPA) software. MetaboAnalyst is a comprehensive web application for metabolomics data analysis and interpretation. Metabolomics pathway analysis used several databases, including the Human Metabolome Database (HMDB; http://www.hmdb.ca/), Metlin (https://metlin.scripps.edu/), and the Kyoto Encyclopedia of Genes and Genomes (KEGG; http://www.genome.jp/kegg/), and the MetaboAnalyst tool, which can identify the most significantly changed metabolism pathways.

### Molecular network analysis

Previous studies applying proteomics and metabonomics to the livers of chronic unpredictive mild stress (CUMS) mouse models of depression reported disturbed lipid metabolism and immune regulation^22,23^. In the current study, we cross-analyzed the two mouse models of depression using IPA. IPA is an advanced bioinformatics software program used to analyze biological pathways and functions of biomolecules of interest. The higher the score, the more relevant the molecules are to the network. The score is calculated using the right-tailed Fisher’s exact test and is based on hypergeometric distribution.

## Results

### Behavioral tests

Results of the behavioral tests are reported in our previous research^9^. Briefly, immobility times for the forced swimming test and the tail suspension test significantly increased and the center motion distance for the open-field test significantly decreased in “depression microbes” mice compared with “healthy microbes” mice, only behavioral significant changes mice used for further analysis. Using FMT, we constructed a mouse model of depression.

### Metabolites showing a significant difference between MDD and CON mice

**Fig. 1:**
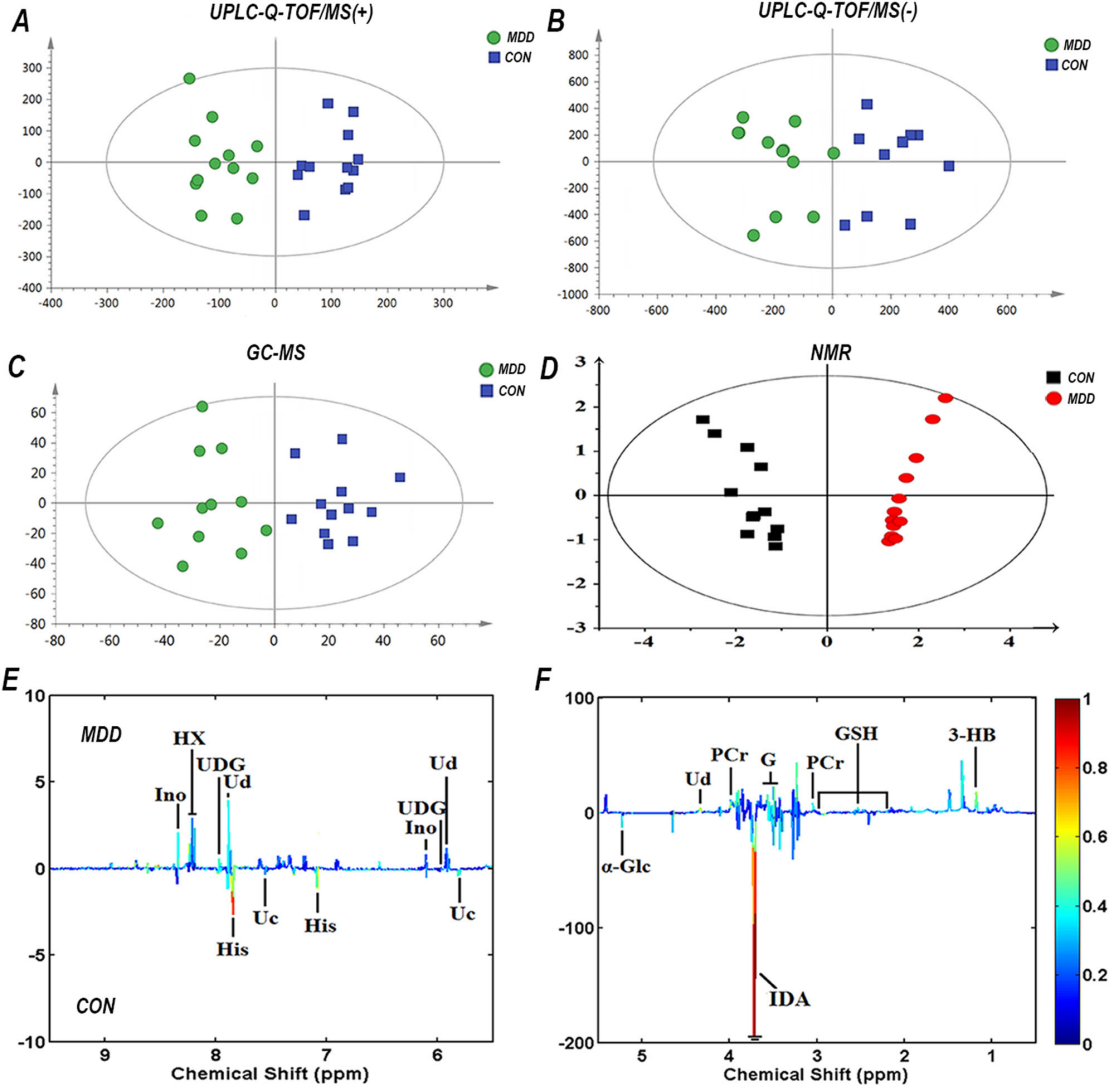
Orthogonal partial least-squares discriminant analysis (OPLS-DA) score plots and ^1^H nuclear magnetic resonance (NMR) corresponding coefficient loading plots. **a**–**c** OPLS-DA score plots derived from ultra-performance liquid chromatography–tandem mass spectrometry (UPLC-Q-TOF/MS) electrospray ionization (ESI) (+), UPLC-Q-TOF/MS ESI (−), and gas chromatography–mass spectrometry (GC–MS) spectra of the major depressive disorder (MDD) group and control (CON) group. **d** OPLS-DA score plots derived from ^1^H Carr–Purcell–Meiboom–Gill NMR spectra of liver extracts and corresponding coefficient loading plots **e**, **f** obtained from the CON group and the MDD group. **e**, **f** Show the significance of metabolite variations between the two classes. Peaks in the positive direction indicate metabolites that are more abundant in MDD. Metabolites more abundant in the CON group are shown as peaks in the negative direction. The key to assignment is shown in Supplementary Fig. 1

Metabolites from 12 MDD and 12 CON mice were used for OPLS-DA analysis. OPLS-DA score plots showed distinct separation between MDD mice and CON mice using the three metabolomics approaches (LC–MS_pos:R2Y = 0.868, Q2 = 0.683; LC–MS_neg:R2Y = 0.798, Q2 = 0.618; GC–MS: R2Y = 0.549, Q2 = −0.694; NMR: R2Y cum = 0.936, Q2 = 0.917) (Fig. 1). R2Y is the cumulative model variation in Y, and Q2 is the cumulative predicted variation. Values for these parameters approaching 1.0 indicate a stable model with predictive reliability. In the current study, R2Y and Q2 values indicated significant metabolic differences between MDD and CON mice. The original total ion chromatograms, typical base peak intensity chromatograms, and ^1^H Carr–Purcell–Meiboom–Gill NMR spectra are shown in Supplementary Fig. 1. A 199-iteration permutation test confirmed that OPLS-DA models were not over-fitted and were valid (Supplementary Fig. 2).

From OPLS-DA analysis, a total of 191 significantly different metabolites were identified between the MDD and CON mice using the three metabolomics approaches (106 decreased and 85 increased in the livers of MDD mice compared with CON mice). Details of the metabolites are shown in Table 1 and Supplementary Table 1.

**Table 1:** Metabolites identified in livers extracts

Metabolite/super class	VIP^a^	FC	r^b^	HMDB	Platform	*P*-value^c^	Trend^d^
Aliphatic acyclic compounds							
Ethanolamine	2.60	–	–	HMDB00149	NMR	–	↓
Trimetlylamine oxide	2.63	–	–	HMDB00925	NMR	–	↓
Phosphocholine	1.89	–	–	HMDB01565	NMR	–	↑
Urea	3.85	1.76	–	HMDB00294	GC–MS	0.04	↑
Putrescine	1.16	1.25	–	HMDB01414	GC–MS	0.03	↑
Amino acids, peptides, and analogs							
Alanine	1.46	–	–	HMDB00161	NMR	–	↑
Glycine	1.08	–	–	HMDB00123	NMR	–	↑
Glycerol	1.79	–	0.64	HMDB00125	NMR	–	↑
Hypoxanthine	–	–	0.68	HMDB00157	NMR	–	↑
Histidine	–	–	−0.90	HMDB00177	NMR	–	↓
Lysine	1.56	–	–	HMDB00182	NMR	–	↑
Phosphocreatine	–	–	0.59	HMDB01511	NMR	–	↑
Iminodiacetate	36.22	–	−0.98	HMDB11753	NMR	–	↓
Alanine	5.44	1.24	–	HMDB00161	GC–MS	0.00	↑
Proline	3.20	1.25	–	HMDB00162	GC–MS	0.01	↑
Isoleucine	2.93	1.45	–	HMDB00172	GC–MS	0.01	↑
Glutamine	3.01	0.46	–	HMDB00641	GC–MS	0.00	↓
Valine	3.80	1.39	–	HMDB00883	GC–MS	0.02	↑
3-Aminoisobutyric acid	4.22	3.21	–	HMDB03911	GC–MS	0.00	↑
Carbohydrates and carbohydrate conjugates							
Glycogen	2.74	–	–	HMDB00131	NMR	–	↑
β-Glucose	1.92	–	–	HMDB00516	NMR	–	↓
Glutathione	–	–	0.58	HMDB00757	NMR	–	↑
α-Glucose	3.55	–	−0.68	HMDB03345	NMR	–	↓
d-Arabitol	1.27	2.19	–	HMDB00568	GC–MS	0.00	↑
Galactinol	14.68	0.59	–	HMDB05826	GC–MS	0.00	↓
Nucleosides, Nucleotides, and Analogs							
Inosine	–	–	0.58	HMDB00195	NMR	–	↑
Uridine diphosphate–glucose	–	–	0.71	HMDB00286	NMR	–	↑
Uridine	–	–	0.61	HMDB00296	NMR	–	↑
Lipids							
Linolenic acid	1.29	1.44	–	HMDB01388	GC–MS	0.03	↑
Oxoproline	6.14	1.35	–	HMDB08177	GC–MS	0.00	↑
Maltotriitol	6.15	0.64	–	HMDB15224	GC–MS	0.00	↓
Organic acids and derivatives							
Lactate	2.42	–	–	HMDB62492	NMR	–	↑
3-Hydroxybutyrate	–	–	0.63	HMDB00357	NMR	–	↑
Lactic acid	3.00	1.14	–	HMDB00190	GC–MS	0.01	↑
Succinic acid	1.56	1.68	–	HMDB00254	GC–MS	0.00	↑
Organophosphorus compounds							
O-phosphorylethanolamine	1.09	0.31	–	HMDB00224	GC–MS	0.01	↓
Phosphomycin	1.85	0.49	–	HMDB14966	GC–MS	0.00	↓
Others/unknown							
Uracil	–	–	−0.56	HMDB00300	NMR	–	↓
Hypoxanthine	3.58	1.29	–	HMDB00157	GC–MS	0.00	↑
Xanthine	2.79	1.27		HMDB00292	GC–MS	0.00	↑
d-(glycerol 1-phosphate)	1.81	0.38	–	HMDB00126	GC–MS	0.01	↓

*VIP* variable importance in projection, *FC* fold change, *HMDB* Human Metabolome Database. All identified metabolites were grouped by super class (based on HMDB website information)

^1^ A VIP value > 1.000 was used as the cutoff value for statistical significance. “-” means the correlation coefficient of │r│ < 0.553 or VIP value of <1.000

^2^ Correlation coefficients: positive and negative signs indicate a positive or negative correlation in the concentrations. A correlation coefficient of │r│ > 0.553 was used as the cutoff value for statistical significance based on discrimination significance at a *p*-level of 0.05 and 11 degrees of freedom (df)

^3^ *p*-Value was derived from two-tailed Student’s *t*-test

^d^ “↑” indicates higher levels in major depressive disorder (MDD), and “↓” indicates lower levels in MDD

### Classification of the significantly changed metabolites

**Fig. 2:**
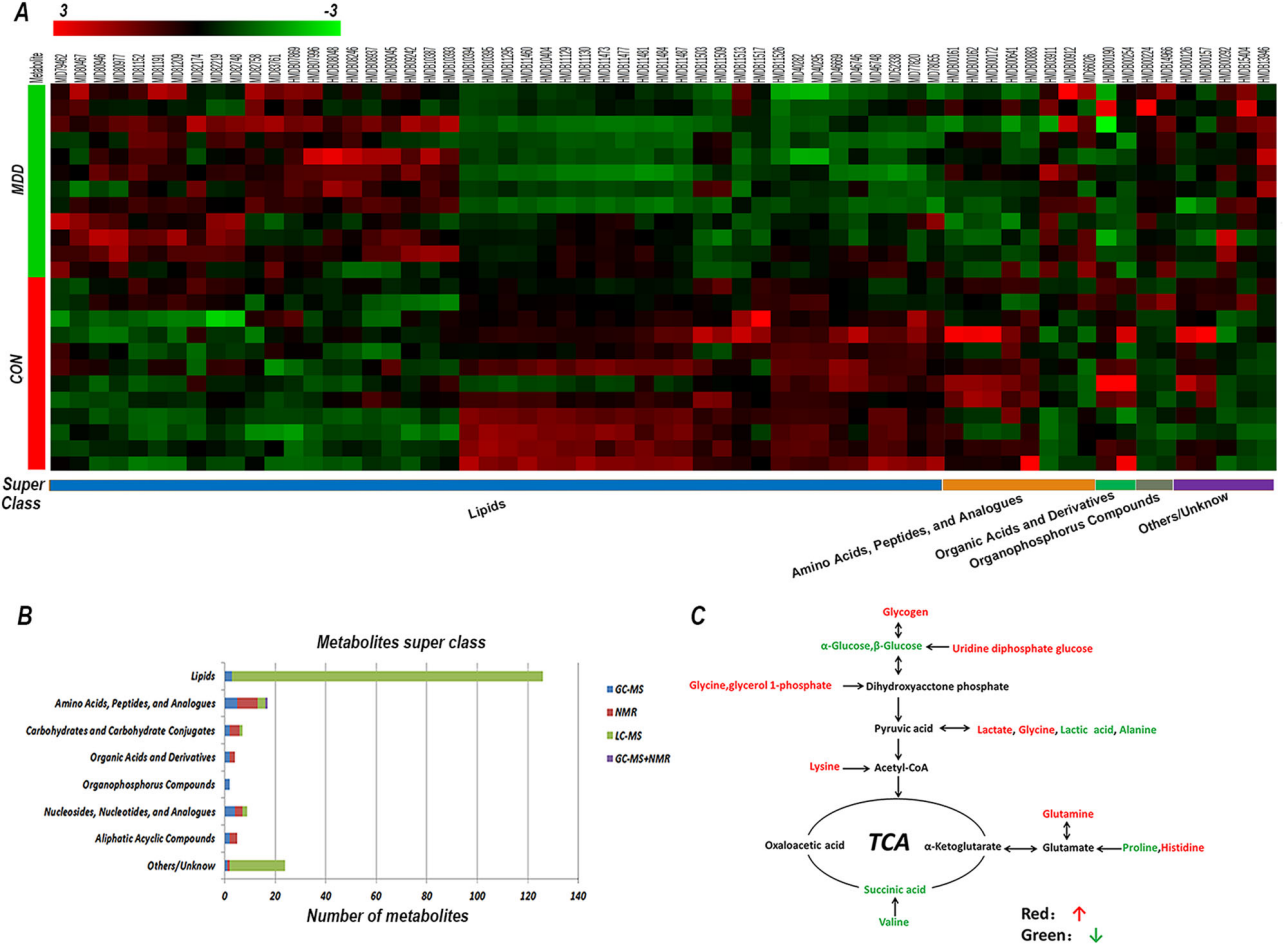
Data on significant metabolites and energy metabolism. **a** Clustering analysis different metabolites in the liver (major depressive disorder (MDD) group vs. the control (CON) group). **b** Number of metabolites identified using the three complementary approaches in each super class. A total of 191 metabolites were identified using gas chromatography–mass spectrometry (GC–MS) (blue), nuclear magnetic resonance (NMR) (red), liquid chromatography–mass spectrometry (LC–MS) (green), or combined approaches (purple), and super classification was performed. **c** Summary of the differential metabolites associated with glycolysis and the tricarboxylic acid (TCA) cycle

Using HMDB and MID for classification of metabolites according to their super class revealed that many belonged to the Lipid super class (linolenic acid, oxoproline, maltotriitol, arachidonic acid, 13-hydroxy-docosanoic acid) and the Amino acids, peptides and analogs super class (alanine, isoleucine, glutamine, valine, iminodiacetic acid), as well as Carbohydrates and Carbohydrate Conjugates (glycogen, glutathione, d-arabitol), Aliphatic Acyclic Compounds (ethanolamine, trimethylamine N-oxide, phosphocholine, urea) among others. A part of metabolites were clustering analyzed and emerged significantly different trends, especially in the Lipid super class (Fig. 2a). Lipid proportion >65% and Amino acids nearly 10% in all metabolites, the number of each class showed in Fig. 2b.

### Metabolites analyzed between FMT and CON mice

The differential metabolites and their respective fold-change were analyzed using MetaboAnalyst, KEGG, and IPA to explore the potential effects of depression microbes. We identified nine pathways with a *p*-value < 0.05 that were different in MDD mice compared with CON mice (Supplementary Table 2). After *p*-values were adjusted using Holm–Bonferroni corrections and the false discovery rate, only glycerophospholipid metabolism significantly changed. Canonical pathway overlapping analyzed using IPA (Supplementary Fig. 3) included tRNA charging, glutamate receptor signaling. A total of 16 metabolites were identified as being significantly associated with glycolysis and the tricarboxylic acid cycle (Fig. 2c).

### System integrated analysis in FMT mice

**Fig. 3:**
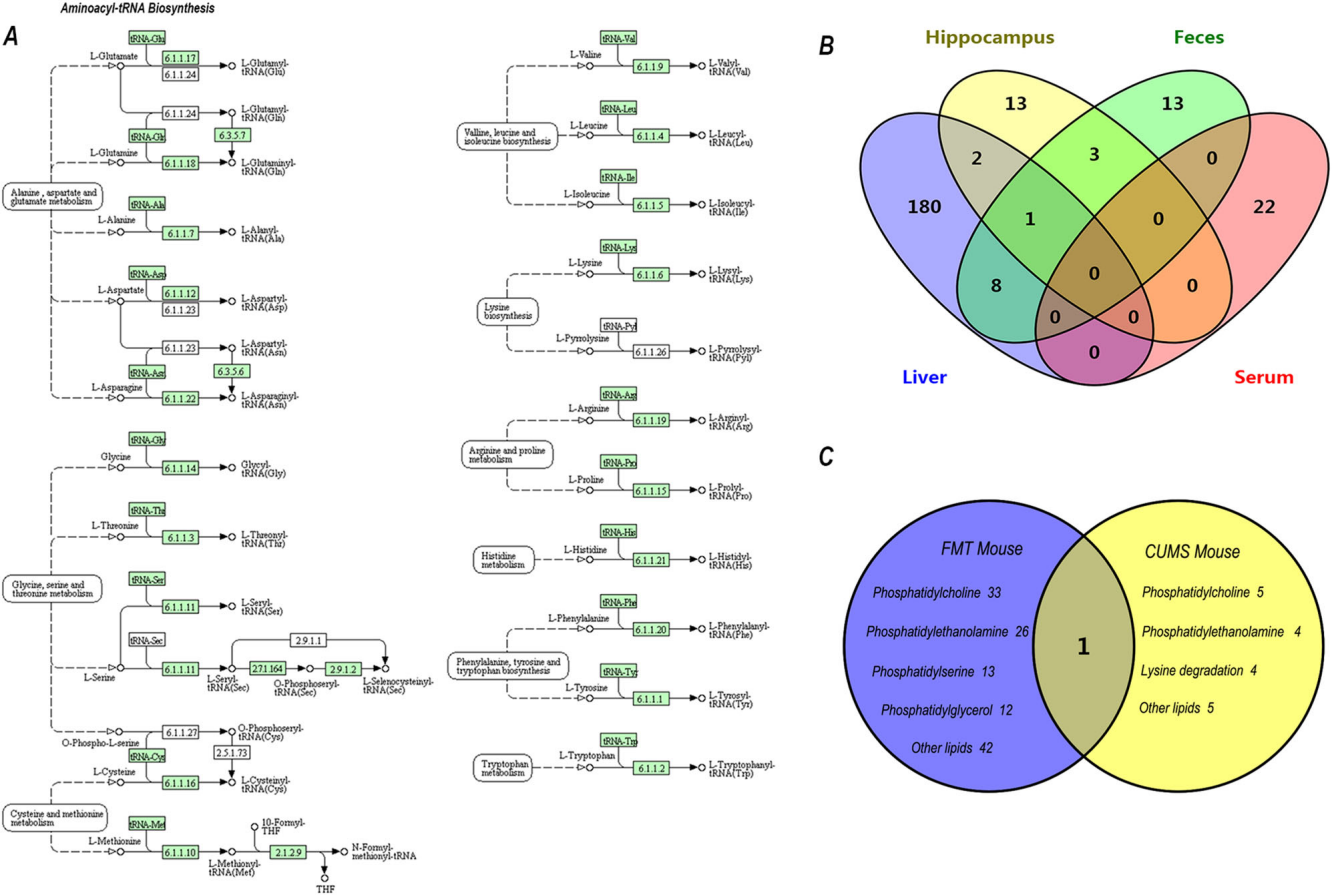
Metabolite cross-talk in different regions and chronic unpredictive mild stress (CUMS) mouse model of depression. **a** Construction of the aminoacyl-tRNA biosynthesis metabolism pathway in mice. The map was generated using the reference map from Kyoto Encyclopedia of Genes and Genomes (KEGG) (http://www.genome.jp/kegg/). Green boxes show enzymatic activities. **b** Venn diagram indicating the number of significant metabolites in different parts of major depressive disorder (MDD) mice. **c** Venn diagram indicating the number of significant metabolites in the livers of the fecal microbiota transplantation (FMT) and CUMS mice models of depression. A common metabolite was hypoxanthine

From system integrated analysis of significant metabolites in the feces, serum and hippocampal samples identified in a previous study^9^, the amino acids asparagine, glutamine, isoleucine, proline, leucine, and glycine, which are involved in aminoacyl-tRNA biosynthesis, were most significantly altered. Details of the KEGG pathways are shown in Fig. 3a.

We also compared overlapping metabolites in different regions of FMT mice (Fig. 3b), and found that nine metabolites in the feces had a close association with the liver, and are mainly involved in energy and lipid metabolism (Supplementary Fig. 4).

### Combined analysis of FMT and CUMS mice

Metabolites showing significant changes detected by LC–MS in livers from FMT and CUMS mice with minimum overlapping are listed in Fig. 3c. In super class, mainly of the metabolites belong to lipid. We analyzed metabolic pathways and glycerophospholipid metabolism was disturbed in FMT mice. Interesting to note that CUMS mice had the same change.

Glycerophospholipid is usually subdivided into phosphatidylethanolamine (PE), phosphatidylcholine (PC), phosphatidic acid (PA), and phosphoinositides (PS). PC species not only protects cells and their organelles from oxidative stress, but also is an essential component of biomembranes. PE species has been identified as modulator of inflammation^24^. The disturbance of glycerophospholipid metabolism indicated oxidative stress, inflammatory cell membrane damage, and even apoptosis in the liver during FMT and CUMS. The common pathway disturbance in liver may play an important role in depression.

### Molecular network analysis of FMT mice using IPA

**Fig. 4:**
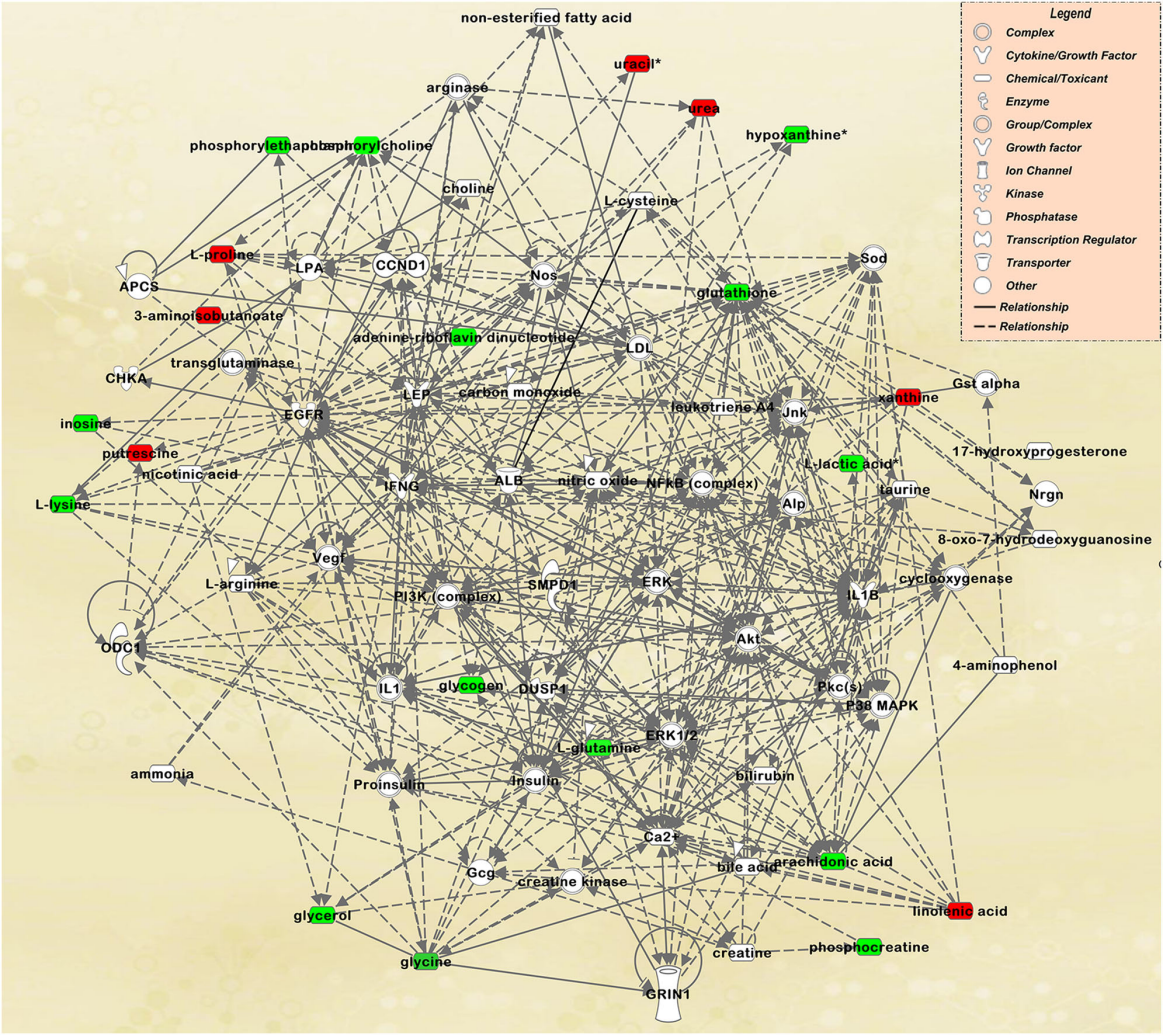
The most significantly changed network between major depressive disorder (MDD) and control (CON) groups. Metabolites in red were upregulated while those in green were downregulated in MDD mice. Solid lines show direct physical interactions (such as binding) between the two parties. Dotted lines show indirect interactions or regulations between the two parties

A total of 191 metabolites and their respective fold-changes were subjected to molecular interaction network analysis using IPA software. Lipid Metabolism, Small Molecule Biochemistry, and Cellular Compromise were the most significantly changed network. A total of 21 metabolites, including l-glutamine, linolenic acid, lysine, phosphorylcholine, urea, l-proline, and glycogen, were associated with the network (Fig. 4).

### Molecular network analysis of FMT and CUMS mice by IPA

In a study that undertook quantitative proteomics analysis of livers from the CUMS mouse model of depression, a total of 66 proteins were reported to exhibit significantly different expression^22^.

We combined proteomics and metabolomics using IPA to examine the two models of depression, which showed behavioral changes stemming from different mechanisms. The Lipid Metabolism, Free Radical Scavenging and Molecule Transports network had a high score of 99, and included high-density lipoprotein, low-density lipoprotein, the nuclear factor κB signaling pathway (Supplementary Fig. 5). From canonical pathway analysis, we found that the overlap centered on Glycogen degradation and Tryptophan Degradation (Supplementary Fig. 6).

## Discussion

Depression is a widespread and debilitating mental disorder that contributes to increased suicide rates and has a heavy socioeconomic burden; however, little is known about its pathogenesis. The gut microbiota is the largest ecosystem in the body and affects numerous physiological functions. The microbe–gut–brain axis is a communication system that integrates neural, hormonal, and immunological signals and metabolites between the gut and the brain^25^. Bacterial products, including lactic acid, organic acids, tryptophan, and propionic acid, have been shown to influence behavior in animals^26^, conjugated fatty acids, LPS, peptidoglycan, acylglycerols, sphingomyelin, and cholesterol can affect intestinal permeability and activate the intestine–brain–liver–neural axis to regulate glucose homeostasis^27^.

Research on depression usually focuses on the central nervous system and peripheral nervous system, rather than the liver. To the best of the authors’ knowledge, this is the first study to apply untargeted metabolomics approaches to the liver of a FMT mouse model of depression.

Liver has a unique vascular system and its blood mainly comes from intestine through the portal vein^28^. Due to the system, liver is vulnerable to exposure to bacterial products. Despite profound interindividual variability, Gram-negative bacteria, such as *Bacteroidetes, Enterobacteriaceae, Alistipes*, and *Proteobacteria* were strongly increased in MDD patients compared with the healthy individuals^29^. Furthermore, increased LPS from Gram-negative bacteria induced endothelial hyperpermeability and “leaky gut” in MDD patients^30,31^. The “leaky gut” increased translocation of gut-derived bacterial products and then stimulated innate immune system, which may involve in the pathophysiology of MDD^32^. In this study, FMT from MDD patients may change gut permeability, hence, liver was exposed to bacterial products and presented disturbed metabolism profile.

In this study, we used three complementary techniques in our untargeted metabolomics approach, these being GC–MS, NMR, and LC–MS. Using these techniques, 191 differential metabolites were distinguished between mice livers treated with “depression microbes” and “healthy microbes”. Importantly, just one metabolite (alanine) was identified by two approaches. The application of complementary approaches to metabolomics for the characterization of liver metabolism is of value. The metabolites identified included: (1) lipids (5-oxoproline and linolenic acid) and lipid metabolism-related molecules (α-glucose, β-glucose, and glycerol); (2) amino acids (glycine, proline, valine, isoleucine, lysine, and histidine); and (3) other metabolites (O-phosphorylethanolamine, putrescine, and trimethylamine N-oxide).

The amino acids, including glutamine, glycine, lysine, valine, and isoleucine, which had significantly changed in the mouse model of depression compared with CON mice. Some of these amino acids have an important role in brain function. Glutamine is a neurotransmitter that plays a crucial role in glutamatergic neurotransmission through contact with astrocytes and neurons in the glutamine–glutamate cycle^33^. Preclinical research has reported that glutamine deficiency in the prefrontal cortex and cerebellum increased depressive-like behavior^34,35^. We found that glutamine significantly decreased in peripheral blood mononuclear cells and the cerebellum of the mouse model of depression, suggesting that it could be a potential biomarker of depression^35–37^. We also conducted metagenomics using murine cecum feces from the same batch samples in this study, and relative abundance of the glutamate biosynthesis enzyme commission numbers showed a contrary trend^9^. The decrease in glutamine may suggest that microbes can affect liver glutamate levels and may modulate depressive-like behavior. The lysine level increased in MDD mice compared with CON mice. Recent studies report that lysine may affect neurotransmitters associated with anxiety and stress in the rat^38^, whereas lysine fortification reduces anxiety and lessens stress in humans^39^. Lysine is a constituent of the serotonin receptor 4 antagonist, which can reduce the level of blood cortisol and the microbe–gut–brain stress response in pigs^40^. In previous studies, we found that lysine levels decreased in the serum of MDD patients^41^, and in the cerebellum and prefrontal cortex of CUMS mice^35,42^. Accordingly, increasing lysine may combat depressive behavior and improve a person’s emotional status.

Metabolism research reports that valine levels decrease in peripheral blood mononuclear cells and the serum of drug-naïve MDD patients^14,43^. Similarly, alanine was reported to decrease in the serum of MDD patients, and may be a potential urine biomarker for MDD patients^44^. In the current study, valine, isoleucine, and alanine increased in MDD mice liver. Levels of the three amino acids showed no difference among the serum, hippocampus, and feces^9^. These results suggest that MDD patients and FMT mice accompanied amino-acid metabolism disturbed and the trends were not exactly same in different parts. The liver is the center for substrate and energy metabolism. Glucose is an important energy provider, and in the current study, it was found to be decreased in MDD mice compared with CON mice. Other metabolites involved in glycolysis and the tricarboxylic acid cycle were markedly downregulated, such as lactic acid (Fig. 4). Combined IPA and canonical pathway analysis revealed glycogen degradation. These changes indicate a disturbance in energy metabolism. In agreement with previous research, a deficiency in circulating glucose was observed with serum and urine metabolomics studies of MDD patients^14,44^. Also, levels of glucose were markedly decreased in the prefrontal cortex of LPS-induced mice^17^. Blass et al. reported two clinical cases in which patients with symptoms of depression showed significant improvement in mood after 2 weeks administration of supplemental malic acid and glucose^45^. Zheng et al. conducted a metabolomics analysis of feces and serum using the same FMT mouse model of depression and found increased levels of carbohydrate metabolites in MDD mice^9^. Combining previous results with the results of the current study suggest that “depression microbes” may lead to a glucose disorder in liver. As the brain consumes 25% of the total glucose available in the body, the decrease in liver glucose may result in depressive behavior.

Phosphocreatine is a high-energy phosphate compound abundant in the central nervous system and functions as a transporter in cell energy exchange. It can transfer high-energy phosphate to ADP to provide ATP, generating creatine. Creatinine is a non-enzymatic by-product of creatine and phosphocreatine. In the current study, phosphocreatine showed a significant increase in the livers of MDD mice compared with CON mice. Zheng et al. reported that creatine in the serum of MDD patients was significantly decreased^14^. We did not detect the secondary metabolite creatinine in the current study, but metabolism research has reported that creatinine in the urine of MDD patients and in the cerebellum of CUMS mice decreased^29,44^. Upregulated phosphocreatine in the liver may be a compensatory energy source, providing beneficial help to improve depressive behavior.

The findings suggest that disturbances to glycolysis and the tricarboxylic acid cycle and the phosphocreatine–ATP pathway support previous research suggesting that a disturbance in energy metabolism may participate in the pathophysiology of depression, and the liver may play an important role.

Disturbances to oxidative stress are reported to be associated with the pathogenesis of MDD^46^. In the current study, we found that the metabolites glycine and glutathione were significantly upregulated in MDD mice. These metabolites are involved in oxidative stress. As glutathione is the primary free radical scavenger in the brain, lower glutathione levels compromise central nervous system anti-oxidative activity^47^. Additionally, lower levels of glutathione and oxidative damage could constitute early signaling events in cell apoptosis^48^. Glycine has been suggested to be a member of glutathione biosynthesis. In our previous metabolism research using the same FMT mouse model of depression, we found that glutathione levels decreased in the prefrontal cortex and the cecum, and glycine decreased in the hippocampus^9^. The metabolites in different parts may show different trends in diseased conditions. The elevated levels of glutathione in the liver suggest increased anti-oxidation activity, which may provide protection from oxidative stress in MDD mice.

Disturbances in lipid metabolism have been reported to be associated with geriatric depression in elderly patients^49^ and in rodent models of depression^50^. In the current study, lipid-related molecules (a total of 126; 66% of all metabolites) showed a tendency to change in the livers of MDD mice. These molecules included O-phosphorylethanolamine, glycerol, and arachidonic acid. Glycerol is the final product of triglyceride metabolism. These findings suggest that MDD mice may have lipid metabolism dysregulation. Combined proteomics of CUMS mice livers showed a high score for the Lipid Metabolism, Free Radical Scavenging and Molecule Transports network. Furthermore, common fecal and liver metabolites appeared to show disturbed lipid metabolism. Disturbances in the metabolism of the three major nutrients in the MDD liver may account for the high comorbidity between MDD and metabolic syndrome^51^.

This study has some limitations. The findings and conclusions drawn need to be treated cautiously because of the risk for overestimation with the relatively small sample size. Second, we did not validate quantities and species of microbes. The potential mechanism behind the association between microbes and liver metabolism disturbance is unclear. Further, data were obtained from naive mice, additional studies should use an adult animal model of depression and conduct in vitro studies. Finally, we integrated information about metabolites trends from different depressive models, organs, and regions as far as possible. However, the potential relationships are not clear and need further research. In future study, we will focus on the mechanism of single strains inducing liver metabolism disturbance and the relationship between gut microbiota and depression.

## Conclusion

In this study, we developed and analyzed a FMT mouse model of depression, and the findings contribute to previous studies on the involvement of the gut microbiota in psychiatric disorders. Using GC–MS, NMR, and LC–MS, we identified 191 changed metabolites in the MDD mouse liver compared with CON mice. The study mainly focused on three major disturbances in metabolism, these being associated with lipid, amino acid, and energy metabolism. Combined analyses with proteomics of CUMS mice livers showed a changed lipid network, suggestive of lipid disorders in the livers of mouse models of depression. Conjoint analyses of metabolites in different parts of the FMT model suggested that aminoacyl-tRNA biosynthesis significantly changed and metabolites in feces were most closely associated with the liver. The findings suggest that “depression microbes” can disturb the liver and different parts of the body carrying out metabolic functions, and help determine the relationship between the liver, microbes, and depression. It is possible that treatment targeting microbes may be potential therapies for MDD.

## Electronic supplementary material

Supplementary Fig 1

Supplementary Fig 2

Supplementary Fig 3

Supplementary Fig 4

Supplementary Fig 5

Supplementary Fig 6

Supplementary Table 1

Supplementary Table 2

Supplementary Figure legends
