# Supplementary figures and images for: Metabolite identification in fecal microbiota transplantation mouse livers and combined proteomics with chronic unpredictive mild stress mouse livers
Source: Transl Psychiatry. 2018 Jan 31;8:34. doi: 10.1038/s41398-017-0078-2 (PMC5802540; doi:10.1038/s41398-017-0078-2)

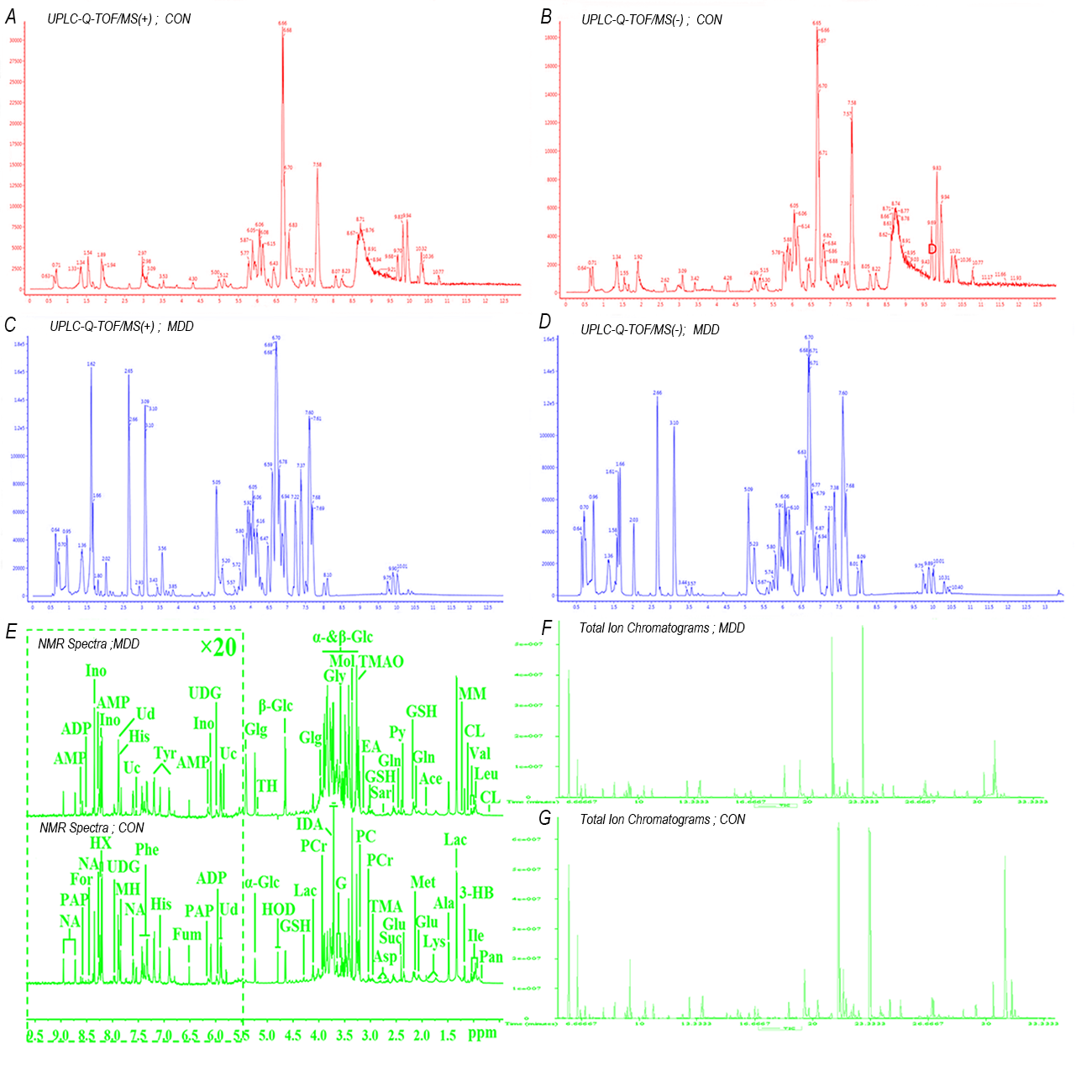

Supplement: Supplementary file 1 — Supplementary Fig 1 [file 41398_2017_78_MOESM1_ESM.tif]

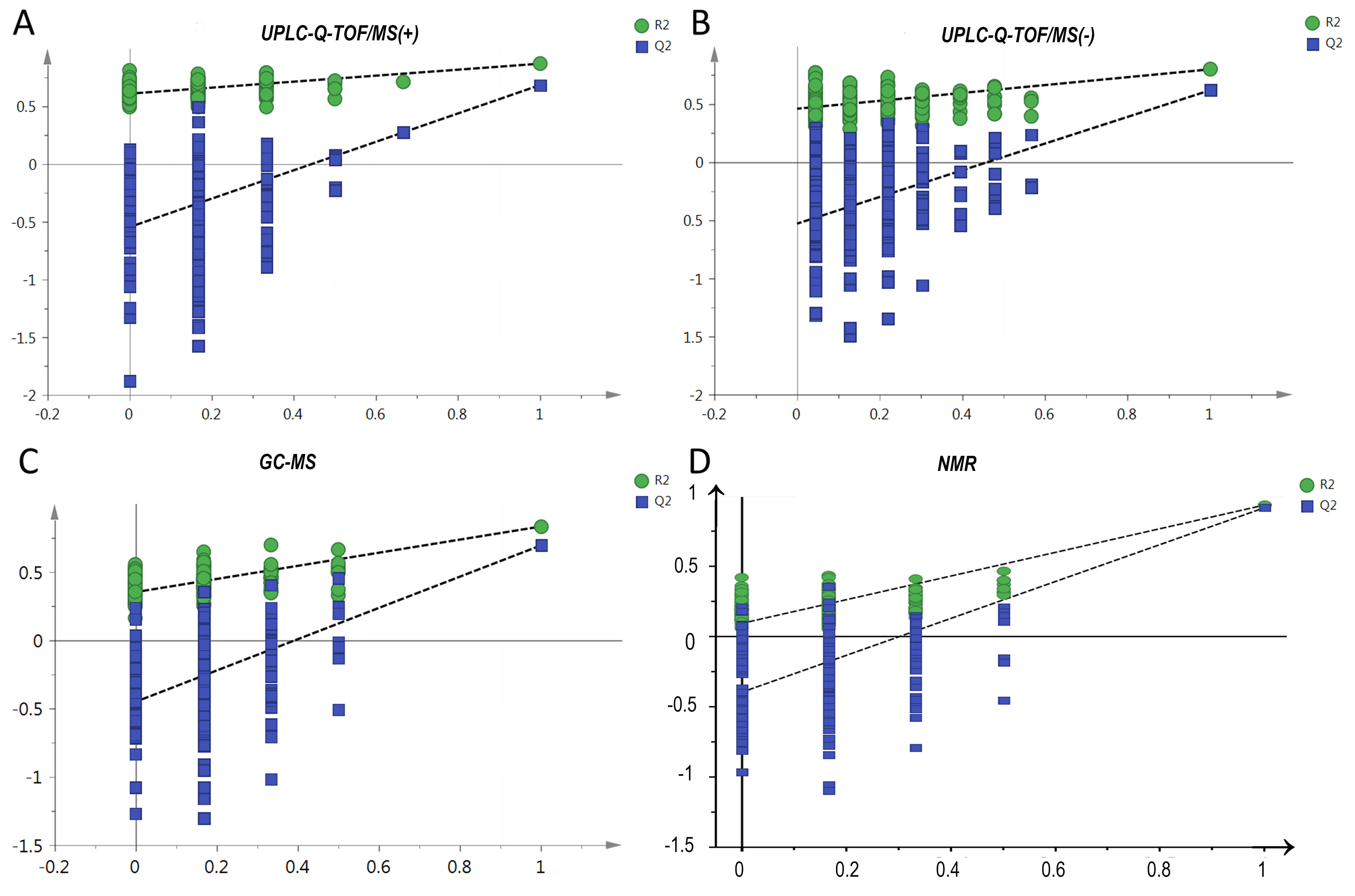

Supplement: Supplementary file 2 — Supplementary Fig 2 [file 41398_2017_78_MOESM2_ESM.tif]

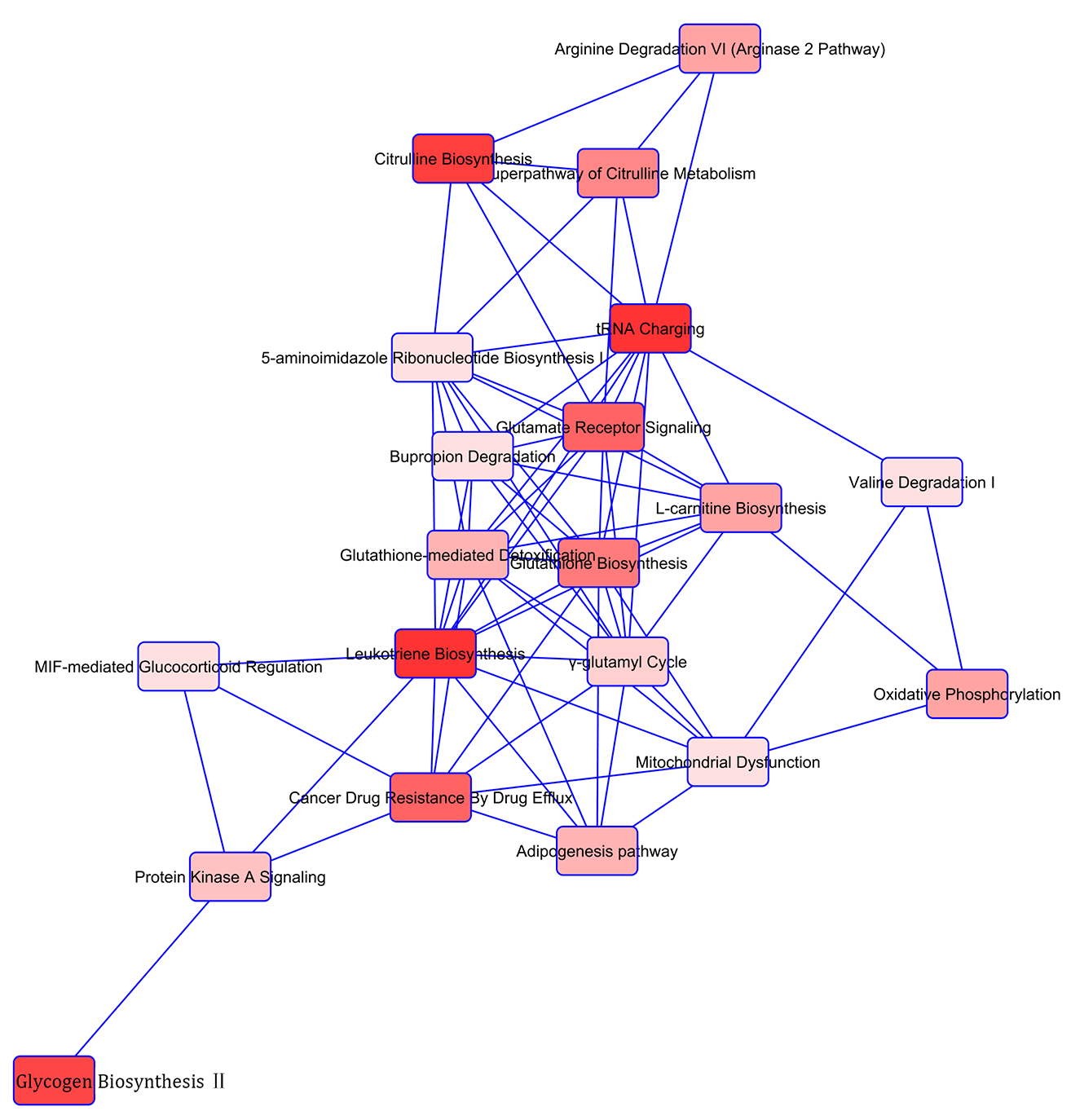

Supplement: Supplementary file 3 — Supplementary Fig 3 [file 41398_2017_78_MOESM3_ESM.tif]

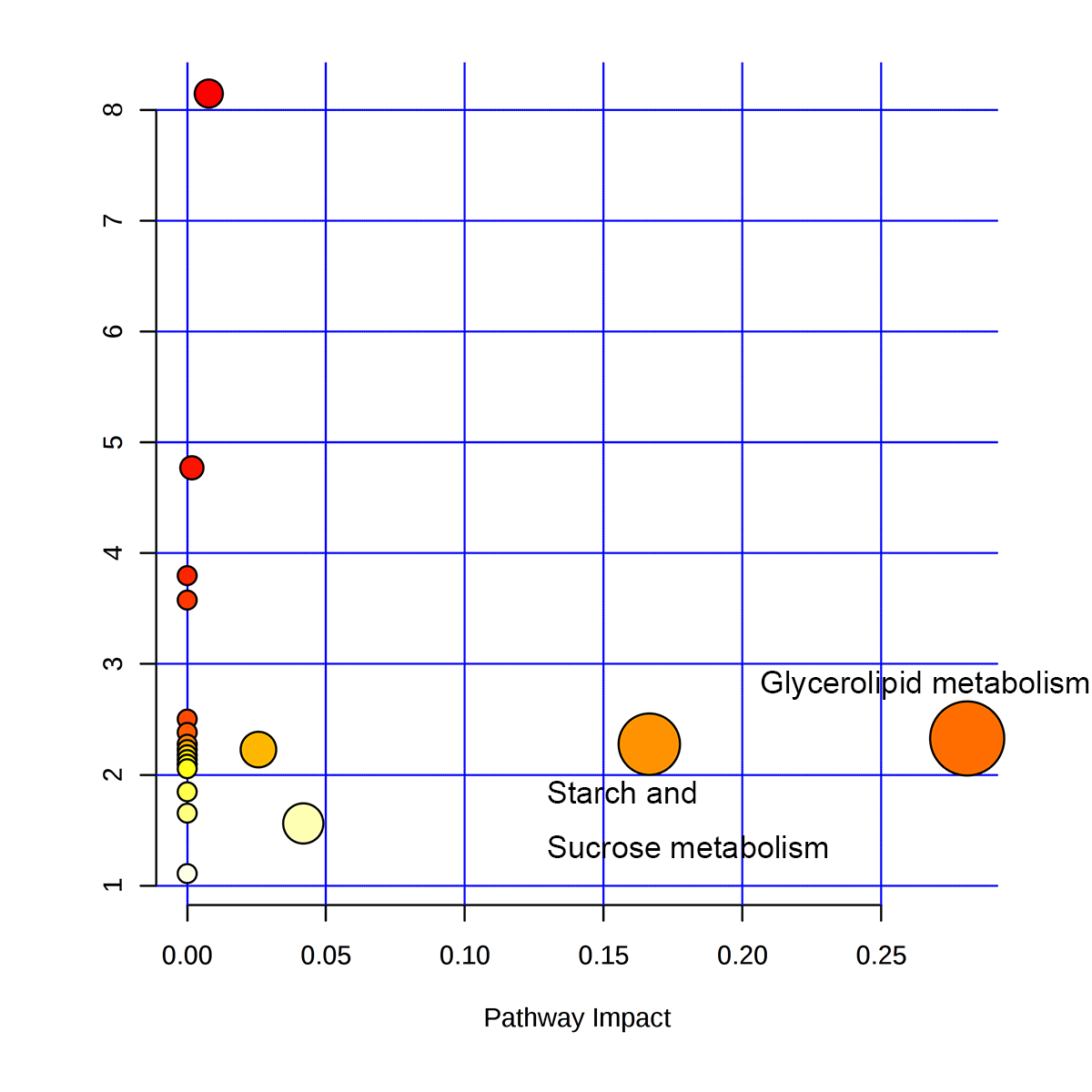

Supplement: Supplementary file 4 — Supplementary Fig 4 [file 41398_2017_78_MOESM4_ESM.tif]

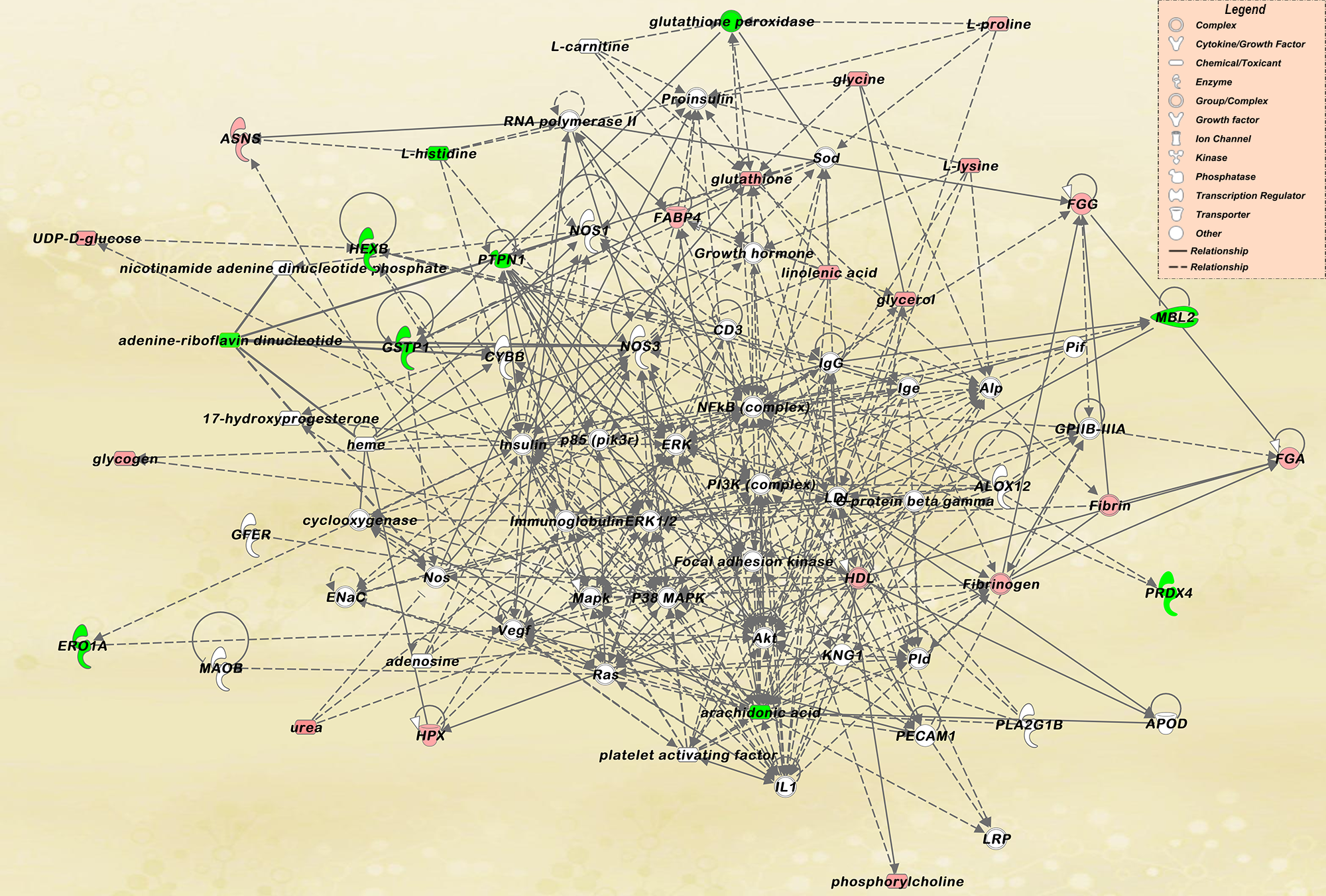

Supplement: Supplementary file 5 — Supplementary Fig 5 [file 41398_2017_78_MOESM5_ESM.tif]

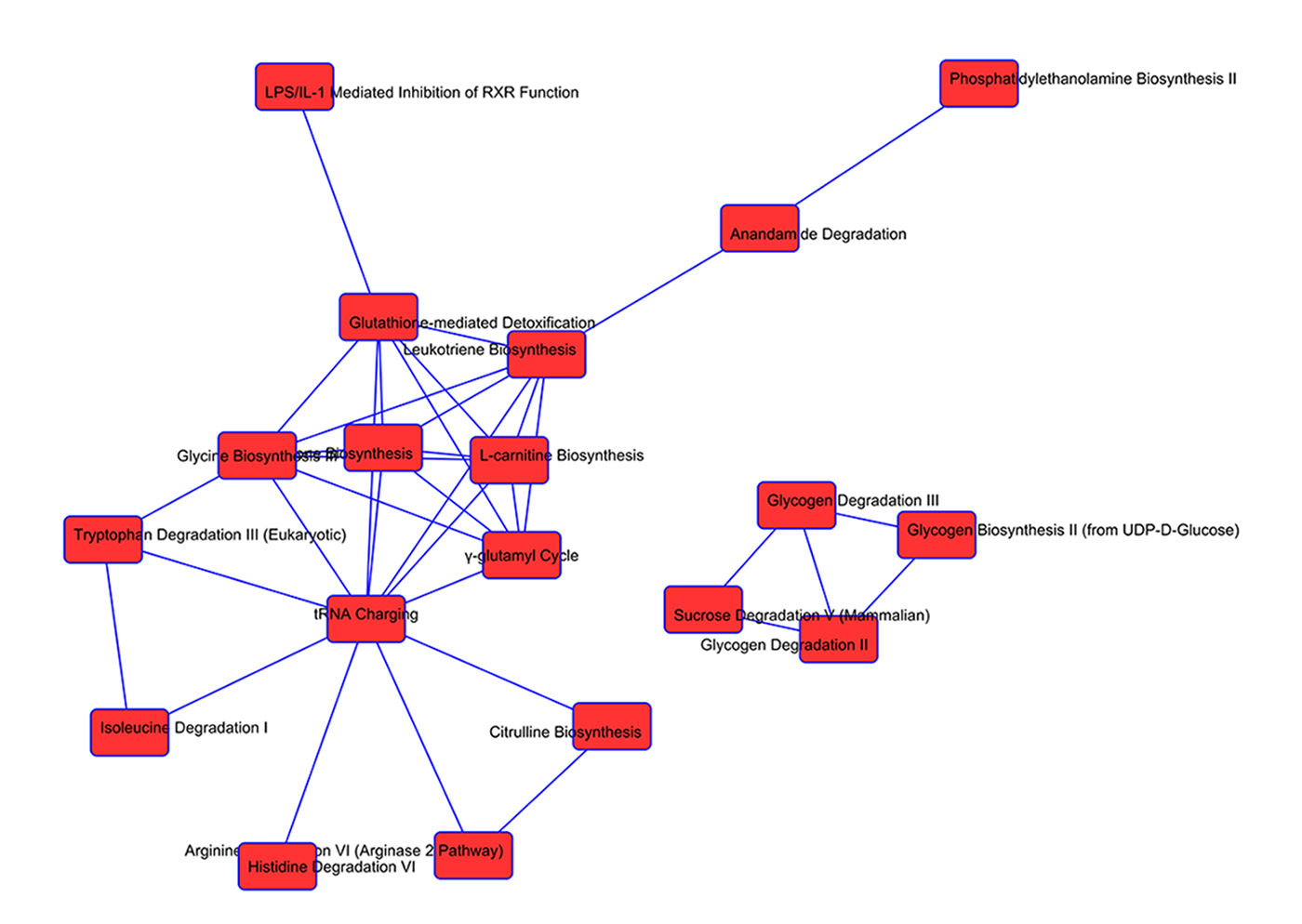

Supplement: Supplementary file 6 — Supplementary Fig 6 [file 41398_2017_78_MOESM6_ESM.tif]
