# Supplementary material for: Metabolite identification in fecal microbiota transplantation mouse livers and combined proteomics with chronic unpredictive mild stress mouse livers
Source: Transl Psychiatry. 2018 Jan 31;8:34. doi: 10.1038/s41398-017-0078-2 (PMC5802540; doi:10.1038/s41398-017-0078-2)
Supplement: Supplementary file 7 — Supplementary Table 1 [file 41398_2017_78_MOESM7_ESM.docx]

**Supplementary Table 1(a)** **Metabolites identified in livers extracts by** **UPLC-Q-TOF/MS ESI (-)**

| Metabolite | VIP | FC^1^ | P-value | m/z | ID |
| --- | --- | --- | --- | --- | --- |
| **Amino Acids, Peptides, and Analogues** | | | | | |
| Succinoadenosine | 1.04 | 2.70 | 0.01 | 382.1 | HMDB00912 |
| N-Adenylyl-L-phenylalanine | 1.95 | 1.99 | 0.04 | 539.14 | MID^2^66026 |
| **Carbohydrates and Carbohydrate Conjugates** | | | | | |
| Dihydrocaffeic acid 3-O-glucuronide | 3.6 | 1.35 | 0.00 | 357.09 | HMDB41720 |
| **Lipids** | | | | | |
| Arachidonic Acid | 3.25 | 1.20 | 0.05 | 303.23 | HMDB01043 |
| 21-Deoxycortisone | 1.15 | 1.23 | 0.02 | 325.18 | HMDB04030 |
| PE(16:0/22:6(4Z,7Z,10Z,13Z,16Z,19Z)) | 1.78 | 1.47 | 0.01 | 762.51 | HMDB08946 |
| LysoPC(18:2(9Z,12Z)) | 3.63 | 1.30 | 0.01 | 564.33 | HMDB10386 |
| LysoPC(22:6(4Z,7Z,10Z,13Z,16Z,19Z)) | 2.96 | 1.32 | 0.01 | 612.33 | HMDB10404 |
| LysoPE(0:0/18:0) | 6.01 | 1.32 | 0.00 | 480.31 | HMDB11129 |
| LysoPE(18:0/0:0) | 1.69 | 1.28 | 0.01 | 480.31 | HMDB11130 |
| LysoPE(0:0/16:0) | 1.65 | 1.35 | 0.00 | 452.28 | HMDB11473 |
| LysoPE(0:0/18:2(9Z,12Z)) | 2.42 | 1.47 | 0.00 | 476.28 | HMDB11477 |
| LysoPE(0:0/20:0) | 1.25 | 1.18 | 0.05 | 554.35 | HMDB11481 |
| LysoPE(0:0/20:3(11Z,14Z,17Z)) | 1.19 | 1.16 | 0.05 | 548.3 | HMDB11484 |
| LysoPC(0:0/20:4(5Z,8Z,11Z,14Z)) | 4.18 | 1.23 | 0.02 | 588.33 | HMDB11487 |
| LysoPE(16:0/0:0) | 5.23 | 1.27 | 0.00 | 452.28 | HMDB11503 |
| LysoPE(18:3(9Z,12Z,15Z)/0:0) | 1.4 | 1.22 | 0.00 | 520.27 | HMDB11509 |
| LysoPE(20:2(11Z,14Z)/0:0) | 2.14 | 1.30 | 0.01 | 504.31 | HMDB11513 |
| LysoPE(20:4(5Z,8Z,11Z,14Z)/0:0) | 4.87 | 1.33 | 0.00 | 500.28 | HMDB11517 |
| LysoPC(18:0/0:0) | 7.28 | 1.23 | 0.01 | 568.36 | MID61694 |
| LysoPC(20:4(5Z,8Z,11Z,14Z)/0:0) | 4.49 | 1.30 | 0.01 | 588.33 | - |
| PC(15:0/0:0) | 4.42 | 1.23 | 0.01 | 480.31 | MID 40282 |
| PC(18:1(6Z)/0:0) | 3.01 | 1.18 | 0.00 | 566.35 | MID 40295 |
| PG(22:6(4Z,7Z,10Z,13Z,16Z,19Z)/  22:6(4Z,7Z,10Z,13Z,16Z,19Z)) | 1.17 | 0.26 | 0.00 | 865.5 | MID 79802 |
| PI(18:0/0:0) | 5.19 | 1.27 | 0.01 | 599.32 | MID 46746 |
| PI(20:4(5Z,8Z,11Z,14Z)/0:0) | 6.36 | 1.27 | 0.00 | 619.29 | MID 46748 |
| PS(15:0/22:0) | 2.08 | 1.59 | 0.01 | 804.58 | MID 77820 |
| PS(18:3(6Z,9Z,12Z)/21:0) | 3.9 | 1.32 | 0.00 | 826.56 | MID 78055 |
| PS(19:1(9Z)/22:4(7Z,10Z,13Z,16Z)) | 2.75 | 1.28 | 0.00 | 850.56 | MID 78173 |
| PS(22:6(4Z,7Z,10Z,13Z,16Z,19Z)/0:0) | 1.81 | 1.64 | 0.00 | 568.27 | MID 78840 |
| PT(18:0/18:1(9Z)) | 3.74 | 1.37 | 0.00 | 802.56 | MID 46669 |
| 1-heptadecanoyl-sn-glycero-3-  Phosphocholine | 3.39 | 1.19 | 0.03 | 508.34 | MID 24068 |
| 1-Linoleoylglycerophosphocholine | 4.73 | 1.37 | 0.00 | 564.33 | MID 1 |
| LysoPE(22:4(7Z,10Z,13Z,16Z)/0:0) | 1.79 | 1.23 | 0.05 | 528.31 | HMDB11523 |
| **Nucleosides, Nucleotides, and Analogues** | | | | | |
| Flavin adenine dinucleotide | 1.17 | 1.16 | 0.01 | 784.15 | HMDB01248 |
| Dephospho-CoA | 2.14 | 1.59 | 0.03 | 686.14 | HMDB01373 |
| **Others/Unknow** | | | | | |
| dihydroergocornine | 1.38 | 1.29 | 0.01 | 608.32 | HMDB15404 |
| Carvedilol(p-Hydroxy) | 1.48 | 2.17 | 0.00 | 403.16 | HMDB13946 |
| 2(α-D-Mannosyl)-D-glycerate | 3.29 | 1.47 | 0.02 | 267.07 | - |
| (25R)-3alpha,7alpha-dihydroxy-  5beta-cholestan-27-oyl taurine | 9.74 | 1.33 | 0.00 | 540.33 | MID57994 |
| Allodesmosine | 1.68 | 1.25 | 0.00 | 636.35 | MID 95178 |
| alpha,beta-Dihydroxanthohumol | 3.25 | 1.89 | 0.00 | 355.16 | MID 52338 |
| Deoxydaunorubicinol aglycone -13-O-b-glucuronide | 1.07 | 2.01 | 0.05 | 541.14 | MID 669 |
| Jurubine | 1.43 | 1.67 | 0.01 | 594.4 | MID 84154 |
| Prenyl arabinosyl-(1->6)-glucoside | 2.64 | 2.27 | 0.00 | 379.16 | MID 95765 |
| Tumonoic Acid I | 1.8 | 1.28 | 0.00 | 542.34 | MID 46669 |
| 6-bromo-heneicosa-5E,9Z-dienoic acid | 3.62 | 1.75 | 0.00 | 381.17 | MID 96826 |

All of the identified metabolites were grouped by super class (based on HMDB and MID). ^1^ Positive values indicate higher levels in MDD subjects, and negative values indicate lower levels in MDD subjects. ^2^ MID, Metlin ID(http://metlin.scripps.

edu/index.php).

**Supplementary Table 1(b) Metabolites identified in livers extracts by UPLC-Q-TOF/MS ESI (+)**

| Metabolite | VIP | FC^1^ | P-value | m/z | ID |
| --- | --- | --- | --- | --- | --- |
| **Amino Acids, Peptides, and Analogues** | | | | | |
| N-palmitoyl tryptophan | 3.33 | 1.05 | 0.00 | 443.33 | MID75477 |
| **Lipids** | | | | | |
| N-Palmitoylsphingosine | 4.44 | 0.59 | 0.00 | 560.5 | HMDB00790 |
| 13-hydroxy-docosanoic acid | 2.66 | 0.53 | 0.00 | 321.31 | HMDB00944 |
| 3-Hexaprenyl-4,5-Dihydroxybenzoic acid | 2.12 | 1.08 | 0.00 | 545.4 | HMDB01063 |
| Cervonyl carnitine | 7.72 | 0.69 | 0.01 | 494.32 | HMDB06510 |
| CDP-DG(18:1(11Z)/18:2(9Z,12Z)) | 2.13 | 0.58 | 0.00 | 986.52 | HMDB06990 |
| PC(14:0/16:0) | 2.44 | 0.29 | 0.00 | 706.54 | HMDB07869 |
| PC(16:0/P-18:1(11Z)) | 3.39 | 0.18 | 0.00 | 744.59 | HMDB07996 |
| PC(18:0/20:4(5Z,8Z,11Z,14Z)) | 5.06 | 0.45 | 0.00 | 810.6 | HMDB08048 |
| PC(18:4(6Z,9Z,12Z,15Z)/20:4(5Z,8Z,11Z,14Z)) | 7.07 | 0.66 | 0.00 | 802.54 | HMDB08246 |
| PE(16:0/20:4(5Z,8Z,11Z,14Z)) | 4.94 | 0.34 | 0.00 | 740.52 | HMDB08937 |
| PE(18:1(11Z)/22:6(4Z,7Z,10Z,13Z,16Z,19Z)) | 3.78 | 0.83 | 0.01 | 790.54 | HMDB09045 |
| PE(20:0/22:5(7Z,10Z,13Z,16Z,19Z)) | 3.63 | 0.39 | 0.00 | 786.59 | HMDB09242 |
| PIP(16:0/18:1(11Z)) | 3.82 | 0.79 | 0.00 | 899.5 | HMDB09924 |
| LysoPC(18:3(6Z,9Z,12Z)) | 10.01 | 0.91 | 0.00 | 518.32 | HMDB10387 |
| LysoPC(20:3(5Z,8Z,11Z)) | 2.36 | 0.74 | 0.01 | 546.35 | HMDB10393 |
| LysoPC(20:3(8Z,11Z,14Z)) | 5.74 | 0.75 | 0.01 | 546.36 | HMDB10394 |
| LysoPC(20:4(5Z,8Z,11Z,14Z)) | 11.07 | 0.76 | 0.01 | 544.34 | HMDB10395 |
| PC(P-18:1(11Z)/22:6(4Z,7Z,10Z,13Z,16Z,19Z)) | 3.82 | 0.32 | 0.00 | 816.59 | HMDB11295 |
| PE(P-18:1(9Z)/22:6(4Z,7Z,10Z,13Z,16Z,19Z)) | 2.31 | 0.21 | 0.00 | 774.54 | HMDB11460 |
| LysoPE(16:1(9Z)/0:0) | 2.35 | 0.44 | 0.00 | 452.28 | HMDB11504 |
| PS(14:0/20:3(8Z,11Z,14Z)) | 2.36 | 0.61 | 0.00 | 758.5 | HMDB12338 |
| PS(16:0/22:6(4Z,7Z,10Z,13Z,16Z,19Z)) | 2.25 | 0.51 | 0.00 | 808.52 | HMDB12362 |
| CDP-DG(16:0/18:1(11Z)) | 2.30 | 1.12 | 0.00 | 962.52 | MID58605 |
| CDP-DG(18:1(11Z)/18:2(9Z,12Z)) | 2.06 | 1.11 | 0.00 | 986.52 | MID58625 |
| DG(18:1(11E)/16:0/0:0) | 4.74 | 1.08 | 0.00 | 559.52 | MID4258 |
| PC(18:0/18:2(10Z,12Z)) | 2.67 | 0.90 | 0.00 | 957.16 | MID39505 |
| LysoPE(18:3(6Z,9Z,12Z)/0:0) | 4.16 | 0.89 | 0.00 | 476.27 | MID62293 |
| PI(18:0/22:0) | 3.82 | 0.85 | 0.00 | 887.64 | MID80977 |
| PI(P-20:0/19:1(9Z)) | 5.70 | 0.83 | 0.00 | 873.62 | MID81152 |
| PS(15:1(9Z)/22:4(7Z,10Z,13Z,16Z)) | 2.18 | 0.83 | 0.00 | 796.52 | MID77854 |
| LysoPE(0:0/20:3(5Z,8Z,11Z)) | 8.39 | 0.82 | 0.00 | 504.31 | MID62273 |
| PE(16:0/22:5(4Z,7Z,10Z,13Z,16Z)) | 4.88 | 0.81 | 0.00 | 766.54 | MID60375 |
| PI(20:0/18:0) | 9.37 | 0.81 | 0.00 | 859.61 | MID80946 |
| PI(16:0/20:0) | 3.60 | 0.80 | 0.01 | 831.57 | MID61170 |
| PC(20:5(5Z,8Z,11Z,14Z,17Z)/22:6(4Z,7Z,10Z,13Z,16Z,19Z)) | 5.68 | 0.80 | 0.01 | 852.55 | MID59964 |
| PC(16:0/18:2(11Z,13Z)) | 3.85 | 0.80 | 0.00 | 758.57 | MID39328 |
| PI(20:5(5Z,8Z,11Z,14Z,17Z)/0:0) | 2.39 | 0.75 | 0.00 | 619.29 | MID81191 |
| PI(19:0/22:6(4Z,7Z,10Z,13Z,16Z,19Z)) | 3.44 | 0.75 | 0.00 | 907.58 | MID80469 |
| PI(16:0/20:0) | 2.12 | 0.74 | 0.01 | 831.57 | MID61170 |
| PC(16:0/18:0) | 4.87 | 0.74 | 0.01 | 762.59 | MID62038 |
| LysoPE(20:4(8Z,11Z,14Z,17Z)/0:0) | 5.45 | 0.74 | 0.01 | 524.27 | MID62302 |
| PS(20:4(5Z,8Z,11Z,14Z)/21:0) | 5.18 | 0.74 | 0.00 | 876.57 | MID78307 |
| LysoPE(20:3(11Z,14Z,17Z)/0:0) | 2.10 | 0.74 | 0.00 | 504.31 | MID62299 |
| 4/20:5(5Z,8Z,11Z,14Z,17Z)) | 2.19 | 0.72 | 0.00 | 804.49 | - |
| PC(16:0/18:4(9Z,11Z,13Z,15Z)) | 4.23 | 0.71 | 0.01 | 754.54 | MID39350 |
| PC(20:4(5Z,8Z,11Z,14Z)/22:6(4Z,7Z,10Z,13Z,16Z,19Z)) | 2.62 | 0.69 | 0.00 | 876.55 | MID59898 |
| PC(18:2(9Z,12Z)/14:0) | 4.21 | 0.69 | 0.00 | 730.54 | MID59576 |
| PI(19:0/22:2(13Z,16Z)) | 2.86 | 0.68 | 0.00 | 971.61 | MID80467 |
| LysoPC(20:2(11Z,14Z)) | 4.30 | 0.67 | 0.01 | 548.37 | MID61702 |
| PC(16:0/16:0) | 7.22 | 0.67 | 0.00 | 734.57 | MID62036 |
| PGF2α isopropyl ester | 3.96 | 0.67 | 0.00 | 361.27 | - |
| PS(19:1(9Z)/20:4(5Z,8Z,11Z,14Z)) | 3.17 | 0.66 | 0.00 | 846.52 | MID78167 |
| PE(18:0/20:2(11Z,14Z)) | 4.34 | 0.66 | 0.00 | 772.59 | MID40522 |
| SM(d18:2/20:1) | 5.09 | 0.65 | 0.00 | 793.56 | MID82758 |
| SM(d18:1/16:0) | 3.12 | 0.64 | 0.01 | 725.56 | MID41586 |
| PC(22:6(4Z,7Z,10Z,13Z,16Z,19Z)/18:4(6Z,9Z,12Z,15Z)) | 2.42 | 0.64 | 0.00 | 826.54 | MID60179 |
| PS(22:6(4Z,7Z,10Z,13Z,16Z,19Z)/19:0) | 4.11 | 0.63 | 0.00 | 872.54 | MID78497 |
| PC(18:3(6Z,9Z,12Z)/20:4(5Z,8Z,11Z,14Z)) | 4.00 | 0.63 | 0.00 | 804.55 | MID59626 |
| PS(19:0/22:4(7Z,10Z,13Z,16Z)) | 2.33 | 0.61 | 0.01 | 876.57 | MID78142 |
| PA(O-18:0/20:4(5Z,8Z,11Z,14Z)) | 3.61 | 0.59 | 0.00 | 711.54 | MID82219 |
| PS(19:1(9Z)/22:6(4Z,7Z,10Z,13Z,16Z,19Z)) | 2.31 | 0.59 | 0.00 | 870.52 | MID78174 |
| PG(18:0/22:5(4Z,7Z,10Z,13Z,16Z)) | 4.37 | 0.58 | 0.00 | 807.57 | MID61884 |
| PG(20:4(5Z,8Z,11Z,14Z)/19:1(9Z)) | 2.80 | 0.57 | 0.00 | 793.54 | MID79462 |
| SM(d18:2/20:0) | 4.54 | 0.55 | 0.00 | 795.57 | MID83761 |
| PC(18:0/18:1(12Z)) | 3.15 | 0.55 | 0.00 | 788.61 | MID39492 |
| PA(22:0/22:6(4Z,7Z,10Z,13Z,16Z,19Z)) | 2.09 | 0.55 | 0.00 | 769.56 | MID81209 |
| PS(19:0/20:5(5Z,8Z,11Z,14Z,17Z)) | 2.19 | 0.53 | 0.00 | 824.55 | MID78137 |
| PC(16:0/18:3(6Z,9Z,12Z)) | 2.59 | 0.53 | 0.00 | 756.55 | MID59423 |
| PC(18:1(9Z)/18:2(6Z,9Z)) | 2.55 | 0.50 | 0.00 | 784.58 | MID39634 |
| PA(O-18:0/18:1(9Z)) | 3.08 | 0.50 | 0.00 | 689.56 | MID82174 |
| PIP(16:0/18:0) | 2.65 | 0.48 | 0.00 | 901.51 | MID61307 |
| SM(d18:2/18:1) | 2.25 | 0.48 | 0.00 | 727.57 | MID82748 |
| PE(18:2(9Z,12Z)/22:6(4Z,7Z,10Z,13Z,16Z,19Z) | 3.92 | 0.48 | 0.00 | 788.53 | MID60518 |
| PE(20:5(5Z,8Z,11Z,14Z,17Z)/22:6(4Z,7Z,10Z,13Z,16Z,19Z)) | 2.76 | 0.46 | 0.00 | 810.51 | MID60869 |
| PA(18:0/20:4(5Z,8Z,11Z,14Z)) | 4.93 | 0.44 | 0.00 | 742.54 | MID40903 |
| PC(O-18:1(9Z)/20:4(5Z,8Z,11Z,14Z)) | 3.79 | 0.41 | 0.00 | 794.60 | MID40018 |
| PIP(16:0/20:3(5Z,8Z,11Z)) | 2.05 | 0.40 | 0.00 | 923.50 | MID61313 |
| N-Stearoyl-D-sphingomyelin | 5.99 | 0.40 | 0.00 | 731.61 | MID438 |
| PE(20:4(5Z,8Z,11Z,14Z)/P-16:0) | 3.26 | 0.38 | 0.00 | 746.51 | MID60807 |
| SM(d18:2/24:0) | 4.97 | 0.31 | 0.00 | 835.67 | MID83781 |
| PE(P-18:1(9Z)/20:4(8Z,11Z,14Z,17Z)) | 5.53 | 0.28 | 0.00 | 750.54 | MID62247 |
| PC(16:0/P-18:0) | 4.68 | 0.24 | 0.00 | 746.61 | MID59443 |
| GlcCer(d18:1/24:0) | 2.58 | 0.20 | 0.00 | 834.68 | MID41612 |
| PIP(16:1(9Z)/18:0) | 2.65 | 0.14 | 0.00 | 889.50 | MID61322 |
| SM(d18:2/24:1) | 2.42 | 0.11 | 0.00 | 833.65 | MID83780 |
| PE(O-16:0/20:4(5Z,8Z,11Z,14Z)) | 2.06 | 0.11 | 0.00 | 726.54 | MID46706 |
| (3beta,5alpha,6beta,24R)-Stigmastane-3,5,6-triol | 3.05 | 1.06 | 0.00 | 487.36 | MID 86665 |
| Vitamin D3 palmitate | 2.75 | 1.04 | 0.00 | 587.55 | MID 42579 |
| 1--2-acetyl-sn-glycerol | 2.38 | 1.04 | 0.00 | 399.31 | - |
| 1-Monopalmitin | 2.42 | 0.84 | 0.00 | 313.27 | MID 24076 |
| O-Arachidonoyl Glycidol | 2.12 | 0.80 | 0.00 | 361.27 | MID 44872 |
| N-Stearoyl-D-sphingomyelin | 5.99 | 0.40 | 0.00 | 731.61 | MID 438 |
| **Others/Unknow** | | | | | |
| 1-arachidonoyl-2-hydroxy-sn-glycero-3-phosphate | 7.55 | 1.20 | 0.00 | 459.25 | MID 265064 |
| Vitamin D3 sulfoconjugate | 4.10 | 1.16 | 0.01 | 487.28 | MID 42578 |
| 13-beta-D-Glucosyloxydocosanoate | 2.12 | 1.04 | 0.00 | 501.38 | MID 66113 |
| Coenzyme Q4 | 8.07 | 0.96 | 0.00 | 455.31 | MID 3909 |
| Feruloyl-diketide-CoA | 2.28 | 0.92 | 0.00 | 986.18 | MID 64198 |
| 3-trans,5-cis-Octadienoyl-CoA | 2.73 | 0.92 | 0.00 | 928.14 | MID 6533 |
| Nicotinate adenine dinucleotide phosphate | 2.08 | 0.90 | 0.00 | 783.04 | MID 4235 |
| N-Cyclohexane carbonyl pentadecylamine | 6.74 | 0.63 | 0.00 | 338.34 | - |
| Cholesteryl-11-hydroperoxy-eicosatetraenoat-e | 3.67 | 0.53 | 0.00 | 705.58 | MID 41724 |
| Arachidonoyl PAF C-16 | 5.21 | 0.31 | 0.00 | 768.59 | MID 43414 |

All of the identified metabolites were grouped by super class (based on HMDB and MID). ^1^ Positive values indicate higher levels in MDD subjects, and negative values indicate lower levels in MDD subjects.
