# Supplementary material for: Metabolite identification in fecal microbiota transplantation mouse livers and combined proteomics with chronic unpredictive mild stress mouse livers
Source: Transl Psychiatry. 2018 Jan 31;8:34. doi: 10.1038/s41398-017-0078-2 (PMC5802540; doi:10.1038/s41398-017-0078-2)
Supplement: Supplementary file 8 — Supplementary Table 2 [file 41398_2017_78_MOESM8_ESM.docx]

**Supplementary Table 2 Result of pathway analysis by MetaboAnalyst**

| Pathway name | Total | Hits^1^ | Raw p^2^ | -log(P) | Holm ^3^ | FDR^4^ | Impact^5^ |
| --- | --- | --- | --- | --- | --- | --- | --- |
| Glycerophospholipid metabolism | 30 | 6 | 3.52E-04 | 7.95E+00 | 2.89E-02 | 2.89E-02 | 3.20E-01 |
| Aminoacyl-tRNA biosynthesis | 69 | 8 | 1.66E-03 | 6.40E+00 | 1.34E-01 | 6.80E-02 | 0.00E+00 |
| Nitrogen metabolism | 9 | 3 | 2.66E-03 | 5.93E+00 | 2.13E-01 | 7.26E-02 | 0.00E+00 |
| Galactose metabolism | 26 | 4 | 1.00E-02 | 4.60E+00 | 7.91E-01 | 1.64E-01 | 3.90E-02 |
| Pantothenate and CoA biosynthesis | 15 | 3 | 1.25E-02 | 4.38E+00 | 9.60E-01 | 1.70E-01 | 1.43E-01 |
| Arginine and proline metabolism | 44 | 5 | 1.45E-02 | 4.23E+00 | 1.00E+00 | 1.70E-01 | 1.68E-01 |
| Alanine, aspartate and glutamate metabolism | 24 | 3 | 4.48E-02 | 3.11E+00 | 1.00E+00 | 3.75E-01 | 1.50E-01 |
| Glutathione metabolism | 26 | 4 | 1.00E-02 | 4.60E+00 | 7.91E-01 | 1.64E-01 | 4.15E-01 |
| alpha-Linolenic acid metabolism | 9 | 2 | 3.48E-02 | 3.36E+00 | 1.00E+00 | 3.56E-01 | 1.00E+00 |

^1^ the actually matched number from the user uploaded data; ^2^ the original p value calculated from the enrichment analysis; ^3^ the p value adjusted by Holm-Bonferroni method; ^4^ the p value adjusted using False Discovery Rate; ^5^ the pathway impact value calculated from pathway topology analysis.
