# Supplementary material for: Metabolite identification in fecal microbiota transplantation mouse livers and combined proteomics with chronic unpredictive mild stress mouse livers
Source: Transl Psychiatry. 2018 Jan 31;8:34. doi: 10.1038/s41398-017-0078-2 (PMC5802540; doi:10.1038/s41398-017-0078-2)
Supplement: Supplementary file 9 — Supplementary Figure legends [file 41398_2017_78_MOESM9_ESM.docx]

**Supplementary figure legends**

**Supplementary Fig. 1** Original chromatograms for each approach

(A and B) Ultra-performance liquid chromatography–tandem mass spectrometry (UPLC-Q-TOF/MS) electrospray ionization (ESI) (+) and UPLC-Q-TOF/MS ESI (−) typical base peak intensity (BPI) chromatograms for the control (CON) group. (C and D) UPLC-Q-TOF/MS ESI (+) and UPLC-Q-TOF/MS ESI (−) BPI chromatograms for the major depressive disorder (MDD) group. (E) ^1^H Carr–Purcell–Meiboom–Gill nuclear magnetic resonance (NMR) spectra (δ0.5–9.5) of liver extracts from the MDD and CON groups. The spectral region δ5.5–9.5 (in dashed box) was magnified 20 times compared with the corresponding δ0.5–5.5 region for clarity. 3-HB, 3-Hydroxybutyrate; Ace: Acetate; ADP: Adenosine diphosphate; Ala: Alanine; AMP: Adenosine monophosphate; Asp: Aspartate; CL: Cholate; EA: Ethanolamine; For: Formate; G: Glycerol; Glc: Glucose; Glg: Glycogen; Gln: Glutamine; Glu: Glutamate; Gly: Glycine; GSH: Glutathione; His: Histidine; HX: Hypoxanthine; IDA: Iminodiacetate; Ile: Isoleucine; Ino: Inosine; Lac: Lactate; Leu: Leucine; Lys: Lysine; Met: Methionine; Mol: Methanol; MM: Methylmalonate; NA: Nicotinamide; Pan: Pantothenate; PAP: Adenosine 3', 5'-diphosphate; PC: Phosphocholine; PCr: Phosphocreatine; Phe: Phenylalanine; Py: Pyruvate; Sar: Sarcosine; Suc: Succinate; TH: Trehalose; TMA: Trimethylamine; TMAO: Trimethylamine N-oxide; Tyr: Tyrosine; U: Unknown; Uc: Uracil; Ud: Uridine; UDG: Uridine diphosphate glucose; Val: Valine. (F and G) Gas chromatography–mass spectrometry (GC–MS) total ion chromatograms (TIC) for the MDD and CON groups.

**Supplementary Fig. 2** Statistical validation of the orthogonal partial least-squares discriminant analysis (OPLS-DA) model by permutation testing

(A) Liquid chromatography–mass spectrometry (LC–MS) positive model, R2=0.609, Q2=−0.547. (B) LC–MS negative model, R2=0.46, Q2=−0.529. (C) GC–MS, R2=0.352, Q2=−0.451. (D) ^1^H nuclear magnetic resonance (NMR), Q2=0.917.

**Supplementary Fig. 3** Canonical pathway overlapping analysis of significantly changed metabolites in livers of major depressive disorder (MDD) mice using Ingenuity pathway analysis (IPA)

**Supplementary Fig. 4** Pathway analysis of common metabolites in major depressive disorder (MDD) mice livers and feces using MetaboAnalyst

**Supplementary Fig. 5** Network of significantly changed proteins in livers from chronic unpredictive mild stress (CUMS) mice and metabolites in livers from fecal microbiota transplantation (FMT) mice

The network mainly involved was the *Lipid Metabolism, Free Radical Scavenging and Molecule Transports* network and had a high score of 99.

**Supplementary Fig. 6** Overlapping canonical pathways for significantly changed metabolites and proteins in the liver
